# Improved Methods of Carnivore Faecal Sample Preservation, DNA Extraction and Quantification for Accurate Genotyping of Wild Tigers

**DOI:** 10.1371/journal.pone.0046732

**Published:** 2012-10-05

**Authors:** Patlolla Anuradha Reddy, Maradani Bhavanishankar, Jyotsna Bhagavatula, Katakam Harika, Ranjeet Singh Mahla, Sisinthy Shivaji

**Affiliations:** Centre for Cellular and Molecular Biology, Hyderabad, India; Institute of Molecular Genetics IMG-CNR, Italy

## Abstract

**Background:**

Non-invasively collected samples allow a variety of genetic studies on endangered and elusive species. However due to low amplification success and high genotyping error rates fewer samples can be identified up to the individual level. Number of PCRs needed to obtain reliable genotypes also noticeably increase.

**Methods:**

We developed a quantitative PCR assay to measure and grade amplifiable nuclear DNA in feline faecal extracts. We determined DNA degradation in experimentally aged faecal samples and tested a suite of pre-PCR protocols to considerably improve DNA retrieval.

**Results:**

Average DNA concentrations of Grade I, II and III extracts were 982pg/µl, 9.5pg/µl and 0.4pg/µl respectively. Nearly 10% of extracts had no amplifiable DNA. Microsatellite PCR success and allelic dropout rates were 92% and 1.5% in Grade I, 79% and 5% in Grade II, and 54% and 16% in Grade III respectively. Our results on experimentally aged faecal samples showed that ageing has a significant effect on quantity and quality of amplifiable DNA (p<0.001). Maximum DNA degradation occurs within 3 days of exposure to direct sunlight. DNA concentrations of Day 1 samples stored by ethanol and silica methods for a month varied significantly from fresh Day 1 extracts (p<0.1 and p<0.001). This difference was not significant when samples were preserved by two-step method (p>0.05). DNA concentrations of fresh tiger and leopard faecal extracts without addition of carrier RNA were 816.5pg/µl (±115.5) and 690.1pg/µl (±207.1), while concentrations with addition of carrier RNA were 49414.5pg/µl (±9370.6) and 20982.7pg/µl (±6835.8) respectively.

**Conclusions:**

Our results indicate that carnivore faecal samples should be collected as freshly as possible, are better preserved by two-step method and should be extracted with addition of carrier RNA. We recommend quantification of template DNA as this facilitates several downstream protocols.

## Introduction

Continuous technological advances over the last two decades have made non-invasive sampling a viable option to study various aspects of forensics and population genetics of elusive wild animals [1]. Individual identification through unique genotypes and subsequent estimation of population size are now being taken up in a number of large carnivore monitoring projects [2], [3]. The main advantage of non-invasively collected samples, like faeces, is that they allow a variety of studies on a species without seeing or disturbing the animal. However, low amplification success and high genotyping error rates are two main impediments to the application of faecal DNA analysis to large scale studies. Fewer samples can be identified up to individual level, and number of PCR reactions needed to obtain correct consensus genotypes increase, escalating analysis time and cost [4]. Taberlet *et al*. [5] recommended two replications of each allele for heterozygous loci and seven replications for homozygous loci. In addition to escalating time and cost of the project, this multiple-tube approach also exhausts the finite sample amounts. Therefore prior to taking up large-scale projects on population estimation and monitoring of elusive animals, it is important to determine factors affecting quality and quantity of sample DNA, and to establish efficient protocols of sample collection, DNA extraction and quantification, so as to optimize work vis-à-vis cost, without trading off on the reliability of genotypes.

Conventional spectrophotometric assays lack the sensitivity to measure low quantity DNA samples and neither can they distinguish between the DNA of interest and co-purified prey DNA in carnivore faecal extracts. Nuclear PCR success rate gives little information on quantity of template DNA obtained, and also does not clearly highlight variations across individuals, samples and loci. Alternatively quantitative PCR assays give us accurate inputs on the amount and quality of template DNA obtained [6]. Quantitative PCR assay on non-invasive samples was first described in genetic studies on wild chimpanzees [7], however its use has not been explored so far in felid studies. After ascertaining optimal amounts of template DNA to avoid genotyping errors in felid faecal extracts, we compared and adopted several pre-PCR protocols to advance our non-invasive studies.

As part of a long term study on tiger populations, we are in the process of assembling multilocus microsatellite genotypes from various tiger habitats of India. As we rely heavily on the use of DNA from faeces, this study was undertaken to address the following aspects -

1. Development of a quantitative PCR assay in felids which will accurately measure amplifiable nuclear DNA obtained from faecal samples. This will allow classification of extracts based on DNA concentrations thereby expediting the genotyping process while increasing the reliability of results [7].
2. Estimation of DNA degradation rate in carnivore faecal samples aged in direct sunlight. This would help us determine an accurate time frame for sample collection and efficiently plan suitable collection strategies.
3. Development of a suitable sample preservation method which will better preserve faecal DNA for at least a period of one month. Three preservation methods – desiccation with silica [8], storage in ethanol [8], and short-term storage in ethanol followed by desiccation with silica [9] were compared and tested on experimentally aged feline faecal samples.
4. Modification of the guanidium thiocyanate (GuSCN) - silica method of DNA extraction [10] so as to significantly improve DNA retrieval from faecal samples [11], [12].

## Materials and Methods

### Sample Collection

#### Wild populations

Between January and March 2010 fresh carnivore faecal samples were collected along all roads and trails, and around water sources within Pench Tiger Reserve, Madhya Pradesh and Buxa Tiger Reserve, West Bengal. Samples were collected in two to three sampling sessions with a gap of fifteen to thirty days between two sessions to allow for the deposition of fresh samples. All samples were collected in fresh self-adhesive plastic bags (Ziploc covers) with silica beads and with their geographic locations appropriately recorded. Samples were transported to the laboratory within 15–20 days and stored at −20°C till extraction. Permissions to collect carnivore faecal samples from Pench and Buxa Tiger Reserves were granted by Principal Chief Conservator of Forests and Chief Wildlife Warden of Madhya Pradesh and West Bengal, respectively (Letter no. 3344 dated 25-06-2008, and Letter no.893/26-5(1) dated 12-03-2010).

#### Captive animals

Experiment 1– Fresh faecal samples (<12 hours old) were collected from seven captive tigers and three captive leopards to assess faecal DNA degradation in direct sunlight. These animals are housed at Nehru Zoological Park, Hyderabad and at the Laboratory for the Conservation of Endangered Species, CCMB. DNA was extracted from about 5g of each faecal sample within two hours of collection (Day 1). Subsequently, the remainder amounts of faecal samples were dried in direct sunlight in open petriplates. Approximately 5g aliquots were taken from these samples on days 3, 5, 7 and 9 and extracted.

Experiment 2– Fresh faecal samples (<12 hours old) were collected from five captive tigers and five captive leopards to evaluate the effectiveness of three different preservation methods. 5g aliquots from each of these samples were extracted within two hours of collection (fresh extract). Remaining amounts of faecal samples were dried in direct sunlight in open petriplates for ten days. Equal aliquots of each sample (approximately 5g) were preserved on days 1, 3, 5, 7 and 9 by three different methods – desiccation with silica; storage in ethanol; 24 hour storage in ethanol followed by desiccation with silica. All aliquots were stored at room temperature for fifteen days followed by freezing at -20°C for fifteen days before extraction.

Permission to collect faecal samples of captive animals at Nehru Zoological Park was granted by Director, NZP, Hyderabad. No specific permits were required to collect faecal samples of captive animals at the Laboratory for the Conservation of Endangered Species, CCMB, Hyderabad.

### DNA Extraction

DNA was extracted from both wild and captive samples by modified GuSCN-silica method [10]. Briefly, the surface layer of about 5g of faecal sample was washed with extraction buffer and centrifuged to remove debris. 15µl of glassmilk (QBIOGENE) was added to the supernatant and incubated at room temperature for an hour. The pellet was washed twice with GuSCN wash buffer and thrice with ethanol wash buffer. Resultant pellet was then air-dried and DNA was eluted in 50µl autoclaved distilled water at 56°C. Sets of 10 samples included an extraction control to monitor for contamination.

In the experiment on preservation protocols, aliquots of ten faecal samples (tiger = 5, leopard = 5) were preserved by three different storage methods. Aliquots of six of these samples (tiger = 3, leopard = 3) were extracted with the addition of 4µg of carrier RNA (Qiagen) along with glassmilk while the remaining four samples (tiger = 2, leopard = 2) were isolated without carrier RNA.

A PCR assay with tiger-specific cytochrome *b* primers (TIF and TIR) developed to differentiate tiger faecal samples from those of sympatric carnivores, like leopards, was used to screen all wild samples [13]. PCR products were electrophoresed and only tiger positive samples were subjected to further analysis.

### Quantitative PCR

**Figure 1:**
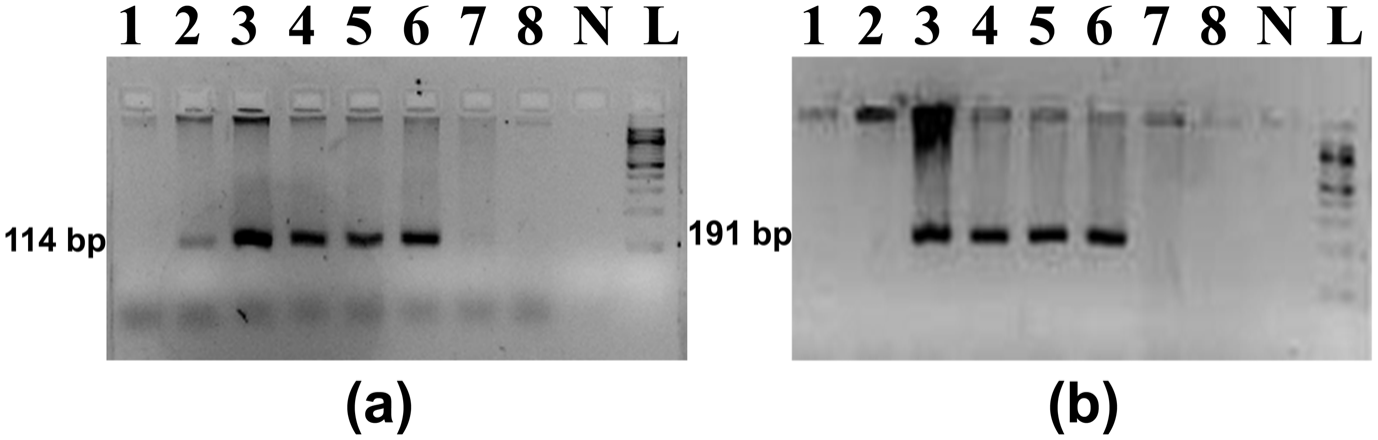
PCR amplification of 114bp and 191bp amplicons of exon 3 of c-*myc* proto-oncogene using two primer sets - (a) CmycEx3-223F and CmycEx3-298R and (b) CmycEx3-71F and CmycEx3-223R respectively. Genomic DNA samples were from Spotted deer (lanes 1 and 2), Asiatic lion (lane 3), Leopard (lane 4), Tiger (lanes 5 and 6), Barasinga (lane 7) and Sambar deer (lane 8). N - Negative control and L - 100 bp ladder.

Two primer pairs (CmycEx3-223F and CmycEx3-298R; CmycEx3-71F and CmycEx3-223R) were specifically designed to amplify 114bp and 191bp of exon 3 of c-*myc* proto-oncogene in domestic cat (Accession no. AF519449), after multiple sequence alignment of above sequence with those of *Bos taurus*, *Sus scrofa*, *Cuon alpinus* (Accession nos. AF519455, AF519454, AF519448). Reference DNA from captive animals (lion, tiger, leopard, and prey species) was used to validate efficacy of both primer pairs to amplify the targeted region only in feline species. Since CmycEx3-71F (5′-CCTTAAGAGATGCCACGTGC-3′) and CmycEx3-223R (5′-TGTGCGTCCGCCTCTTGTCG-3′) amplified 191bp in felids with no corresponding amplification in prey species (Fig.1), this primer pair was used in all further quantitative PCR assays. Amplifications were performed in triplicates with 8µl reaction mixture containing 4µl SYBR Green (Invitrogen), 5pM forward and reverse primers, and 1µl DNA of known concentration for standard curve and 2µl of faecal extracts. PCR was carried out in an ABI 7900 HT Real Time PCR System, with an initial incubation at 50°C for 2 minutes and at 95°C for 10 minutes, followed by 40 cycles of 95°C for 15 seconds, 60°C for 30 seconds and 72°C for 30 seconds. This was followed by a final dissociation cycle of 95°C for 15 seconds, 60°C for 15 seconds and 95°C for 15 seconds. Standard curve was rejected if correlation coefficient of the trendline was <0.95. DNA for the standard curve consisted of 10 dilutions (20ng, 4ng, 800pg, 160pg, 32pg, 6.4pg, 1.28pg, 0.25pg, 0.05pg, 0.01pg and 0.002pg per µl) of tiger blood DNA. 20ng/µl of DNA was initially quantified by absorbance (*A*_260_) in a spectrophotometer and then serially diluted. A no-DNA control was included with each standard curve, and three such controls were included in each plate of faecal extracts. DNA concentrations in the extracts were calculated from slope and Y-intercept (Y_int_) of the trendline from the standard curve, plotted as log of DNA concentrations vs. C_t_ values: DNA concentrations = 10^(*(Ct-Yint)*/slope)^.

Quantitative PCR amplifications were performed in triplicates for each sample. Samples were graded based on number of successful quantitative PCRs. Samples which amplified thrice were grouped into Grade I, samples which worked twice were in Grade II, those which amplified once were in Grade III, and the ones which did not amplify or whose values were undetermined were classified in Grade IV (Table 1).

**Table 1:** Comparative DNA concentrations and PCR success rates of different grades of samples.

Grade	DNA concentration in pg/µl		PCR success at twelve loci (%)
	qPCR	Nanodrop reading	
I	>20	200–45,000	92.34
II	1–20	300–37,400	78.75
III	<1	100–43,000	53.79
IV	Undetectable	150–35,000	–

### Microsatellite Amplification

Tiger DNA extracts from Pench and Buxa Tiger Reserves were genotyped at twelve polymorphic microsatellite loci (F37, F42, F53, Fca96, F115, F124, F141, Fca391, Fca424, Fca441; [14] and E6, E7; [13]). We followed the two-step multiplex PCR assay described by Arandjelovic *et al.,* [15] with modifications. In the initial step, all microsatellite loci were amplified together in a single reaction in triplicates. 15µl PCR mixture consisted of 1XPCR Buffer (*TaKaRa ExTaq* Hot Start version, TaKaRa), 15pM of each primer (unlabelled), 250µM dNTPs, 1X BSA (New England Biolabs), 2U of *Taq* enzyme (*TaKaRa ExTaq* Hot Start version, TaKaRa) and 5µl of template DNA. PCR reactions were carried out in a Mastercycler epgradientS (Eppendorf) with the following conditions: initial denaturation at 95°C for 10 minutes, 40 cycles of 94°C for 15 seconds, 52°C for 20 seconds, 72°C for 30 seconds, followed by a final extension of 72°C for 30 minutes. Triplicate singleplex PCRs at each locus were carried out as above in reaction volumes of 15µl, except that 0.5–0.7µl of the initial multiplex PCR product was used as template instead of DNA extract. PCR mix also contained 5pM each of FAM or HEX fluorescently-labelled forward primer and unlabelled reverse primer. Cycling conditions were also similar as above except for primer-specific annealing temperatures for each singleplex PCR, which varied between 50°C to 62°C. All PCR steps, except the addition of template DNA, were performed in a hood that was UV-irradiated before and after use to avoid contamination. PCR products from the singleplex amplification step were electrophoresed on an ABI 3730 Genetic Analyzer and alleles were sized relative to an internal control (500 LIZ™, Applied Biosystems) using GeneMapper software version 3.7 (Applied Biosystems).

### DNA Degradation and Preservation Protocols

Quantitative PCR using c-myc primers was also performed on experimentally aged felid faecal DNA extracts. Firstly, single factor ANOVA was used to test for individual variation in DNA concentration for the 2 species examined, namely tiger (n = 7) and leopard (n = 3). Trend in DNA degradation due to ageing in direct sunlight was examined with non linear curve fitting. DNA degradation in sunlight, and effect of three preservation methods on DNA quality over time, were analyzed with non-linear regression of non transformed values of DNA concentrations with the first order polynomial equation, y = a+bx, where ‘b’ is the slope and ‘a’ is the intercept. Tukey’s multiple comparison was used to determine differences in DNA yield between three different storage methods. All comparisons were performed with DNA extracted from fresh faecal samples of the same animals before commencement of various treatments. All calculations were done in Microsoft EXCEL spreadsheets. Statistical analysis was performed with PRISM3 software. For all analyses, significance level was measured at less than 0.05.

## Results

### DNA Quantification

The oligonucleotides, CmycEx3-223F and CmycEx3-298R, failed to show specificity for felid samples and repeatedly amplified an 114bp amplicon in some prey species. However CmycEx3-71F and CmycEx3-223R, exclusively amplified a 191bp region of exon 3 of c-*myc* proto-oncogene in the felid species tested with no amplification in prey species (Fig.1). These primers were subsequently used to quantify amounts of amplifiable nuclear DNA present in tiger positive samples collected from two forests of India. Out of a total of 399 faecal samples collected from Pench and Buxa Tiger Reserves, 146 were positively of tiger origin. Following quantitative PCR analysis, these tiger positive samples were graded based on the number of successful quantitative PCRs (Table 1). Average DNA concentration of Grade I faecal extracts was 982pg/µl (20.34–30996.45pg/µl), while Grade II and Grade III had an average of 9.5pg/µl (1.25–19.03pg/µl) and 0.4pg/µl (0.02–0.99pg/µl) respectively. 9.6% of faecal extracts had no amplifiable DNA. All tiger positive DNA extracts were also quantified spectrophotometrically, and concentrations ranged from 0.1 to 45ng/µl irrespective of sample grade (Table 1).

### Microsatellite Amplification from Faecal Extracts

PCR success rate and accuracy of microsatellite amplification were analyzed at twelve loci for 146 wild tiger faecal samples. Genotypes were obtained by two-step multiplex PCR method and all criteria for estimating genotyping errors were as described by Arandjelovic *et al*., [15]. PCR success was calculated as the proportion of positive microsatellite PCRs/total PCRs. PCR success was 92% for samples in Grade I, and 79% and 54% for those in Grades II and III respectively (Table 1). Allelic dropout frequency was calculated for samples in each grade. Table 2 shows that extracts with very low amounts of template DNA (<5pg/reaction) also give substantial number of positive PCRs, but exhibit higher allelic dropouts at many of the loci (7–33%). Data of all loci were combined and dropout rate was used to calculate the number of repetitions needed for homozygous loci to obtain results with 99.99% certainty for samples of different DNA categories (Table 3). Two repetitions were sufficient to reach this level of certainty when template DNA was more than 100pg/reaction (Grade I). Three replications were needed when template concentrations were between 5 and 100pg (Grade II), while four repetitions were necessary to reach this certainty level when DNA concentrations were below 5pg/reaction (Grade III). Results of individual locus showed that some loci could be reliably amplified with two repetitions even when template DNA was below 5pg. However certain other loci needed as many as seven repetitions to achieve the same level of certainty (Table 2). Nevertheless when template DNA was more than 100pg, even these difficult loci could be accurately scored with two replications.

**Table 2:** PCR success rate and dropout per locus for different categories of DNA.

Locus	Grade	PCR success (%)	Dropout (%)
F37	I	89	3
	II	51	3
	III	46	22
F42	I	81	0
	II	63	2.5
	III	37	17
F53	I	97	2
	II	74	7
	III	52	33
Fca96	I	95	2.5
	II	81	5
	III	58	13
F115	I	95	0
	II	95	6
	III	56	13.5
F124	I	100	3
	II	88	5
	III	46	17
F141	I	90	1
	II	67	5
	III	32	12
Fca391	I	86	1
	II	80	3
	III	57	12
Fca424	I	95	0
	II	90	5
	III	68	11
Fca441	I	95	1.5
	II	83	5
	III	80.5	7
E6	I	99	1
	II	97.5	3
	III	72	12.5
E7	I	86	2
	II	73	9.5
	III	41	21

**Table 3:** Number of PCR replicates necessary for various grades of template DNA to obtain genotypes with high confidence (99.99% certainty).

Grade	PCR Success (%)	Drop out (%)	PCR replications needed
I	92.34 (±5.54)	1.53 (±1.07)	2
II	78.75 (±13.05)	4.87 (±1.91)	3
III	53.79 (±13.98)	15.97 (±6.66)	4

### DNA Degradation

**Figure 2:**
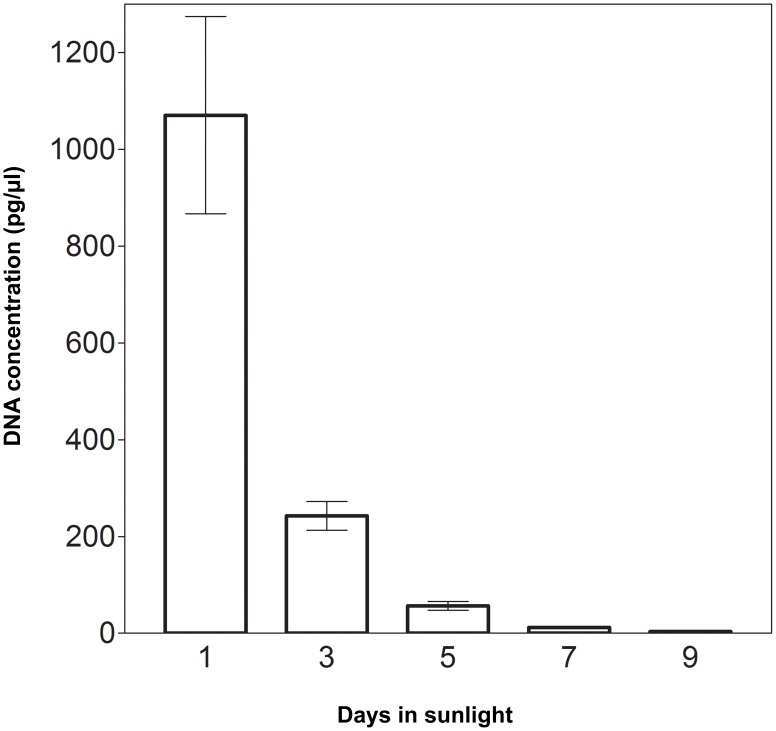
Graph showing DNA degradation rate in 10 faecal samples exposed to direct sunlight. Maximum DNA degradation occurs between Day 1 and Day 3 of exposure to direct sunlight (p<0.001).

DNA quantities in experimentally aged faecal extracts are given in Table 4. Variations in DNA concentrations between individuals were not significant for both tigers (p = 0.73) and leopards (p = 0.8). Variations in DNA concentrations were also not significant when samples from both the species were analyzed together (p = 0.94). Non-linear regression analysis on experimentally aged felid faecal DNA extracts showed a negative trend, indicating that DNA degraded progressively when samples were exposed to direct sunlight (tiger slope = −119; leopard slope = −117.5). Since multiple regression slopes did not differ significantly between the two carnivore species tested (p>0.05), degradation data for tigers and leopards were clubbed together. Regression slope for combined data of tigers and leopards showed a negative trend (slope = −118.3). Results showed that aging of samples in direct sunlight had a significant effect on quantity and quality of amplifiable DNA (p<0.001). Nonparametric Tukey’s multiple comparison between DNA concentrations of Day 1 and Day 3 was highly significant (p<0.001) but those between other time points, i.e. between Day 3 and Day 5, Day 5 and Day 7, Day 7 and Day 9 were not significant (p>0.05). This shows that maximum DNA degradation in faecal samples occurred within 3 days of exposure to direct sunlight (Fig.2).

**Table 4:** DNA concentrations of experimentally aged faecal samples estimated through quantitative PCR.

**Days in direct sunlight**	**DNA conc. (pg/ul) (n = 10)**	**DNA conc. (pg/ul) Tiger (n = 7)**	**DNA conc. (pg/ul) Leopard (n = 3)**
1	1070.9 (±645.6)	1090.2(±813.1)	1051.6(±525)
3	242.7(±95.2)	212.3(±99.9)	273(±89.9)
5	56.9(±28.8)	40.3(±11.7)	73.4(±32.3)
7	11.9(±6.5)	8.1(±2.8)	15.5(±7.2)
9	3.7(±2.8)	2.1(±1.1)	5.4(±3.1)

### Sample Preservation Protocols

**Figure 3:**
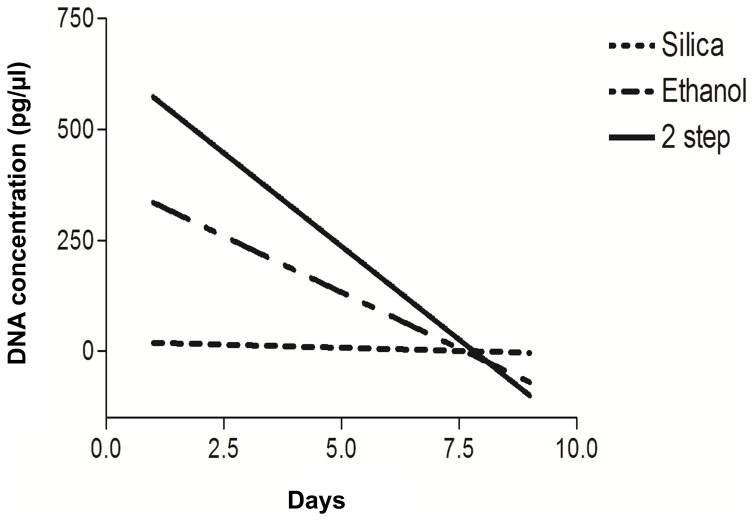
Non linear regression graph of DNA concentrations obtained from samples stored by three preservation methods. Non linear regression slopes for silica, ethanol and two-step methods were -2.8, -50.6 and -84.11 respectively.

Tukey’s multiple comparison showed that Day 1 DNA concentrations of samples preserved by the two-step method were not significantly different from fresh DNA extracts (p>0.05), but the difference was significant for ethanol storage method (p<0.1) and highly significant for silica method (p<0.001) (Fig.3). However by Day 3, DNA concentrations of samples stored by two-step storage method significantly differed from those of fresh extracts (p<0.1) and this difference was highly significant for the other two storage methods (p<0.001). From Day 5 onwards, fall in DNA concentration was highly significant irrespective of the preservation method used (p<0.001). Nevertheless, throughout the experiment period, two-step storage method yielded an average of 2.0- 3.0 times more DNA than silica and ethanol methods (Table S1).

Multiple comparison tests were also done between the three preservation methods to determine which one fared better. DNA concentrations of Day 1 extracts showed that ethanol method was better than silica storage method, but two-step method performed better than both ethanol and silica storage methods (silica v/s ethanol, p<0.01; silica v/s two-step, p<0.001; ethanol v/s two-step, p<0.05). Difference in DNA concentrations of Day 3 extracts was significant only between two-step and silica methods (p<0.05). There was no significant difference between silica and ethanol or ethanol and two-step methods (silica v/s ethanol, p>0.05; two-step v/s ethanol, p>0.05). From day 5 onwards, DNA concentrations were not significantly different (p>0.05) for all three methods.

### Addition of Carrier RNA

In order to determine whether addition of carrier RNA had any significant effect on DNA recovery from faecal samples, we first compared DNA concentrations of fresh faecal samples exposed to sunlight, which were extracted with and without addition of carrier RNA. There was a significant difference in DNA recovery with the addition of carrier RNA for tiger and leopard samples at all time points (Single factor ANOVA, tigers, p = 0.006; leopards, p = 0.02). DNA concentrations in tiger and leopard faecal sample extracts without the addition of carrier RNA were 816.5pg/µl (±115.5) and 690.1pg/µl (±207.1) respectively, while concentrations with addition of carrier RNA were 49414.5pg/µl (±9370.6) and 20982.7pg/µl (±6835.8) respectively.

Next we analyzed whether addition of carrier RNA significantly affected DNA yields from faecal samples stored by three preservation methods. Addition of carrier RNA significantly improved DNA recovery from Day 1 extract stored by two-step method but did not greatly improve DNA recovery from the other two methods (two-step method no carrier RNA Day 1 v/s two-step method carrier RNA Day 1, p<0.001; silica method no carrier RNA Day 1 v/s silica method carrier RNA Day 1, p>0.05; ethanol method no carrier RNA Day 1 v/s ethanol method carrier RNA Day 1, p>0.05). However, from Day 3 onwards, addition of carrier RNA did not significantly improve DNA recovery in any of the three preservation methods (p>0.05).

Multiple comparison tests between the three preservation methods with addition of carrier RNA showed that DNA yield was significant for the two-step method as compared to the other methods on Day 1 (Tukey’s test - Silica Day 1 carrier RNA v/s Ethanol Day 1 carrier RNA, p>0.05; Silica Day 1 carrier RNA v/s two-step Day 1 carrier RNA, p<0.001). However from Day 3 onwards, DNA yield was not significantly better in any of the preservation methods (Tukey’s test - Silica Day 3 carrier RNA v/s Ethanol day 3 carrier RNA, p>0.05, Silica Day 3 carrier RNA v/s two-step Day 3 carrier RNA, p>0.05) (Table S1).

## Discussion

As part of a large-scale DNA study on various wild tigers populations in India, we are in the process of assembling multilocus nuclear DNA genotypes from faecal DNA extracts. Faecal samples are the preferred source of DNA, as these can be obtained easily in relatively large numbers and do not require capture of animals. Difficulties in obtaining reliable genotypes from such samples are mainly due to low quality and quantity of template DNA. Although Taberlet *et al*., [5] recommended two replications of each allele for heterozygous loci and seven replications for homozygous loci, in practice, researchers working with non-invasively obtained low concentration DNA rarely follow the multiple-tube approach completely. Limited amounts of DNA in addition to high cost and efforts are the main constraining factors inhibiting repeated PCRs to obtain correct consensus genotypes. However errors in microsatellite genotypes can have disastrous consequences for studies attempting individual identification or paternity/haplotype assignment, as these studies draw conclusions based on presence of mismatches between individuals [7].

Quantification of template DNA will allow classification of samples based on DNA content and thus help in fastening the genotyping process along with assured reliability of results. Towards this we designed a real-time PCR assay which specifically and accurately measures amounts of amplifiable nuclear DNA of feline origin without amplifying co-purified DNA of prey or other sympatric carnivores. This PCR assay is simple and eliminates the necessity to repeatedly genotype all tiger positive faecal samples collected thereby speeding up the genotyping process without compromising on the reliability of results. Spectrophotometric assays lack the specificity to quantify target DNA, but instead measure total DNA (Table 1). This can be very misleading leading to repeated failed PCRs [7]. We quantified DNA extracted from 146 tiger faecal samples and graded them according to the number of successful quantitative PCRs. The species-specific PCR assay used to identify tiger faecal samples is based on amplifying a 162bp region of mitochondrial cytochrome *b* gene [13]. Many samples positively identified by this assay as tiger samples contained undetectable amounts of nuclear DNA (Grade IV, Table 1). This may be due to relatively higher amounts of mtDNA compared to nuclear DNA and also due to differential stability of the two types of DNA during storage [7]. Murphy *et al*., [4] have shown similar results in faecal extracts of brown bear, where it is generally simpler and more feasible to identify species using mtDNA irrespective of quality/freshness of the sample obtained or preservation method used, while success rates in individual identification using multilocus nDNA genotyping are usually lower.

Following quantitative PCR, DNA extracts were graded, with Grade I starting with a minimum of 20pg/µl or 100pg per multiplex reaction. This corresponds to half the cut-off used in DNA extracts of chimpanzee or gorilla samples [7], [15]. Despite using far lesser amounts of template DNA, results obtained in our study are comparable to those given by Arandjelovic *et al*., [15]. We show a strong relation of PCR success rate to initial amounts of DNA used. Although samples with very less template DNA (<5pg) showed fair PCR success rate (54%), allelic dropout rate was highest (16%) for this category. Approximately one-tenth of the 146 faecal extracts had undetectable amounts of DNA. Accurate quantification of DNA extracts will help us decide protocols and hasten future work. Extracts containing higher amounts of DNA can be replicated lesser number of times, while those with very low DNA content can be repeatedly amplified or discarded. Two-step multiplex method will further help in expediting the genotyping process while saving up on the difficult to obtain samples. We can also infer from our data that there is a clear difference in allelic dropout rate when each locus is analyzed separately and when data of all loci are combined (Tables 2 and 3). Loci like F42 and F141 show lower PCR success rate but exhibit low allelic dropouts when template DNA is less than 5pg. Contrarily F53, F37 and E7 show high dropout rates when this category of DNA is used. Future work in various wild populations can be planned after quantifying all samples and genotyping subsets of each DNA grade, results of which can then be used to plan protocols for the remaining samples.

Our study on experimentally aged faecal samples of leopards and tigers shows that maximum degradation happens within three days of defaecation (Fig.2). Similar results were reported earlier in brown bear and wolf [3], [4]. Piggott [16] reported rapid faecal DNA degradation in both herbivore and carnivore species. Ageing has been reported as the worst of several factors which degrade faecal DNA [3], [17], [18]. Low amounts of template DNA can severely affect PCR success rates and also increase allelic dropout rates and occurrence of false alleles. Although considerably difficult, sample collection programs should be planned to facilitate collection of fresh faecal samples at frequent intervals. This will increase the number of samples which yield better and more DNA thereby increasing our chances of getting reliable genotypes.

We also compared three different storage methods [9] to determine the best preservation method which could better preserve faecal samples till extraction. The two-step preservation method is able to retain DNA quality and quantity in fresh samples (not more than three days old at the time of collection) for a storage period of at least one month (Fig.3). However this is not the case when fresh samples are collected in ethanol or stored with silica beads. This promise of substantially higher success rates in the laboratory should offset all logistical difficulties of collecting samples by the two-step method. Better sample condition means fewer samples need to be collected and fewer PCRs are needed to obtain reliable results. The only precaution to be taken here is careful handling during transfer of samples from ethanol to silica to avoid contaminations and sample mix-up. Addition of carrier RNA during the extraction protocol further improves DNA recovery from freshly collected faecal samples (Table S1) thereby enhancing our chances of generating reliable genotypes of wild tigers of India.

Based on this study we conclude that faecal samples should be collected as fresh as possible and should be stored by the two-step method. DNA should be extracted from these samples as soon as possible without prolonged storage and preferably with the addition of carrier RNA at the time of extraction. We strongly recommend accurate quantification of template nuclear DNA as this step will facilitate several downstream protocols. Repeated DNA extractions would no longer be required as a single DNA extract from a sample would be sufficient for genotyping that sample by the two-step multiplex protocol. Prior to taking up a large scale study based on faecal DNA samples, small subsets of graded DNA could be amplified at different loci and based on the results obtained, guidelines could then be decided for accurate genotyping of the remaining samples.

## Supporting Information

Table S1 DNA concentrations for tiger and leopard faecal samples extracted with and without addition of carrier RNA and stored by different preservation protocols.
(XLS)
